# Mitral repair for moderate ischemic mitral regurgitation in patients with end-stage ischemic cardiomyopathy

**DOI:** 10.1016/j.xjon.2026.101963

**Published:** 2026-07-10

**Authors:** Yusuke Misumi, Daisuke Yoshioka, Shin Yajima, Takuji Kawamura, Ai Kawamura, Kazuma Handa, Akinobu Ohtani, Shunsuke Saito, Kazuo Shimamura, Shigeru Miyagawa

**Affiliations:** Department of Cardiovascular Surgery, The University of Osaka Graduate School of Medicine, Osaka, Japan

**Keywords:** ischemic mitral regurgitation, ischemic cardiomyopathy, mitral valve repair, coronary artery bypass grafting, left ventricular reverse remodeling

## Abstract

**Objectives:**

For patients with end-stage ischemic cardiomyopathy undergoing coronary artery bypass grafting (CABG), the indications for concomitant repair of moderate mitral regurgitation (MR) remain controversial. This study evaluated outcomes of concomitant mitral valve repair (MVr) for moderate ischemic MR in patients with advanced left ventricular (LV) remodeling and severely impaired LV function undergoing CABG.

**Methods:**

We retrospectively reviewed 341 patients (mean age, 67 ± 9 years) with ischemic cardiomyopathy (LV ejection fraction, ≤40%) and less-than-severe MR who underwent CABG. Of these, 108 (32%) underwent concomitant MVr for clinically relevant moderate MR, and 233 (68%) underwent CABG alone for mild or absent MR. All patients had a prior myocardial infarction. The primary end point was overall survival. Secondary end points included postoperative changes in MR grade and LV function at 1 year. Median follow-up was 4.5 years (interquartile range, 2.4-7.3 years).

**Results:**

Despite higher preoperative surgical risk in the CABG + MVr group (European System for Cardiac Operative Risk Evaluation II score, 6.7% vs 2.9%; *P* < .001), 30-day mortality did not differ between groups (1.9% vs 2.1%; *P* = .88). Five-year overall survival was 73% versus 77% (log-rank *P* = .25). At 1 year, the CABG + MVr group demonstrated greater LV reverse remodeling (median LV end-diastolic volume index reduction: −26% vs −19%; *P* = .001), higher LV ejection fraction (46 ± 13% vs 42 ± 12%; *P* = .044), and a lower prevalence of significant MR (6% vs 13%; *P* = .032).

**Conclusions:**

In patients with advanced ischemic cardiomyopathy and moderate MR, concomitant MVr can be performed with acceptable operative risk and promotes superior LV reverse remodeling. Long-term survival was comparable to patients with nonsignificant MR who did not require mitral intervention, suggesting that targeted mitral repair may normalize the adverse prognostic influence of moderate ischemic MR.

## Graphical Abstract

**Figure:**
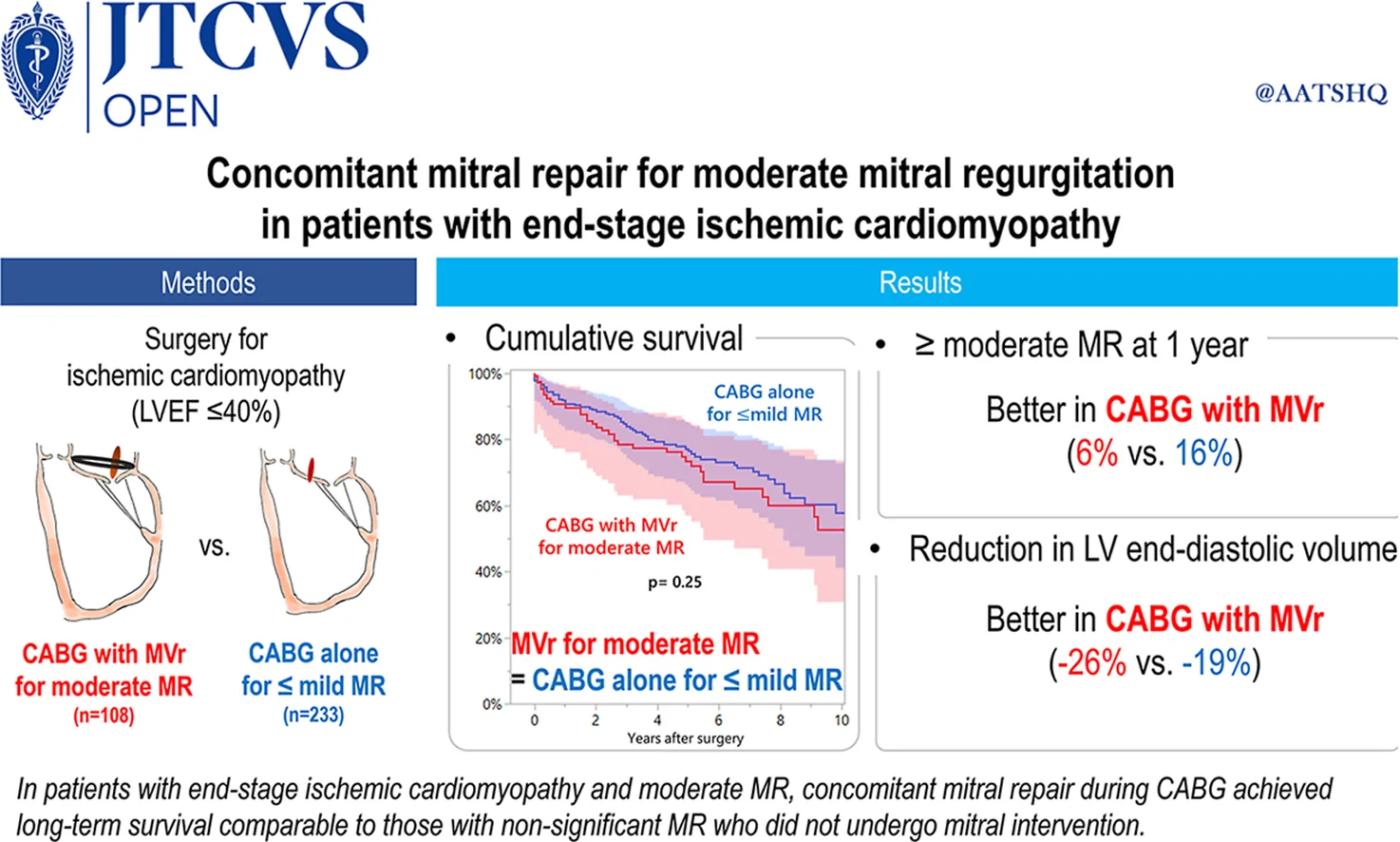

**Figure:**
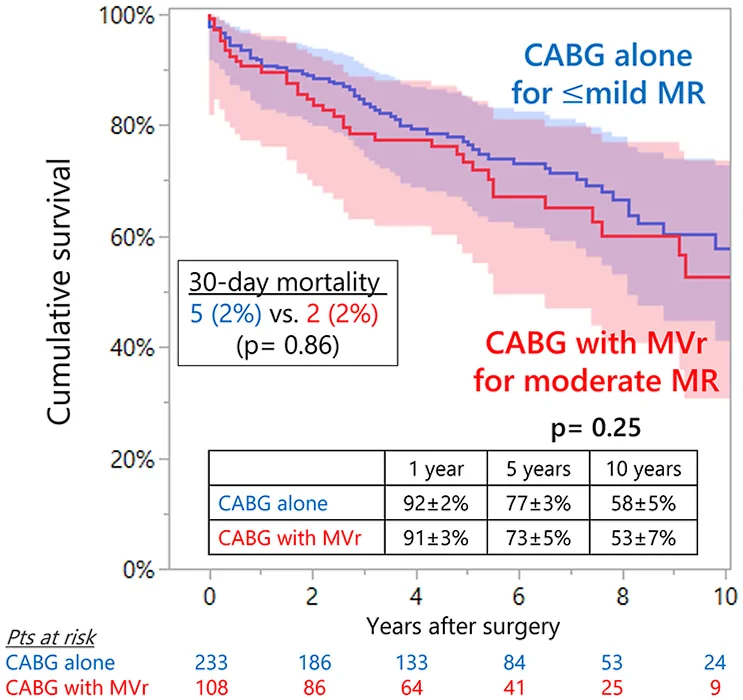
Mitral repair with CABG normalizes prognosis in end-stage ischemic cardiomyopathy with MR.

**Central Message**

In end-stage ischemic cardiomyopathy with moderate MR, adding mitral repair to CABG is safe, improves LV remodeling, and matches survival of patients with mild or no MR.

**Perspective**

The best treatment for moderate ischemic MR in patients with severely reduced LV function is still debated. This study shows that adding mitral repair to CABG is safe, drives greater LV reverse remodeling than CABG alone for mild or no MR, and yields similar long-term mortality. These findings support a more aggressive surgical strategy for moderate ischemic MR when advanced LV remodeling is present.

In patients with coronary artery disease and left ventricular (LV) dysfunction, ischemic mitral regurgitation (MR) is an established adverse prognostic factor for both heart failure and overall survival.^1^, ^2^, ^3^, ^4^ Among patients with reduced ejection fraction (EF), even mild MR has been associated with a worse prognosis compared with its absence.^5^, ^6^, ^7^ Although surgical intervention on the mitral valve is generally accepted when severe MR is present at the time of coronary artery bypass grafting (CABG), the indications for concomitant mitral surgery in cases of moderate MR remain controversial.^8^^,^^9^

Several observational studies and randomized controlled trials have evaluated the clinical efficacy of concomitant mitral valve (MV) surgery for moderate MR during CABG.^10^, ^11^, ^12^, ^13^, ^14^, ^15^, ^16^, ^17^, ^18^ These studies suggest that combined mitral surgery may provide benefits with respect to postoperative relief of heart failure symptoms, improvement in LV function, and reduction of MR severity. However, assessments of long-term survival outcomes have been limited, and many enrolled patients had relatively preserved LV function. Consequently, evidence regarding the influence of concomitant MV surgery on long-term outcomes in patients with severely impaired LV function remains scarce.

We hypothesized that in patients with ischemic cardiomyopathy, concomitant MV repair (MVr) with CABG would normalize the adverse prognostic influence of moderate ischemic MR. To test this hypothesis, we compared long-term survival between patients undergoing CABG with MVr and those with only mild or absent MR.

## Patients and Methods

### Patients

This retrospective study included 341 patients with ischemic cardiomyopathy (LVEF ≤40%) who underwent CABG with (n = 108) or without (n = 233) concomitant MVr for less-than-severe MR between 1995 and 2023. All the patients had LV systolic dysfunction and dilation caused by ischemia with a documented prior myocardial infarction more than 30 days after onset. Patients selected for mitral surgery had clinically significant MR (grade 3+) at maximum symptom burden, attributable to restricted leaflet closure. Exclusion criteria included degenerative mitral disease, concomitant aortic valve surgery, surgical ventricular restoration, or emergency operations. A flow diagram depicting patient selection is provided as Figure E1. Patients’ data were retrieved from the Osaka Cardiovascular Research Group database, approved by the institutional ethics committee of Osaka University Hospital; written informed consent was waived given the retrospective design.

### Surgical Procedures

In the CABG-alone group, the choice between off-pump and on-pump technique was determined according to the patient's clinical profile— including cardiac function and aortic lesions—and surgeon preference. In patients undergoing CABG with MVr, conventional cardiopulmonary bypass with mild systemic hypothermia was used. Myocardial protection was achieved by combined antegrade and retrograde cold blood cardioplegia. All patients in the MVr group underwent stringent restrictive mitral annuloplasty following careful assessment of the inter-commissural distance and anterior leaflet height. The choice of ring size was left to the discretion of the individual surgeon. Papillary muscle approximation was performed in 6.2% of the MVr group at the surgeon's discretion. No additional procedures were performed on the mitral valve itself.

### Assessment of LV Function and MR Severity

Standard 2-dimensional and Doppler echocardiography was performed preoperatively and at 1 year after surgery. Additionally, in the CABG + MVr group, MR grade was also assessed at 3 years after surgery. MR was graded semiquantitatively as 0 (absent), 1+ (trivial), 2+ (mild), 3+ (moderate), or 4+ (severe) based on color Doppler jet extent and spatial distribution relative to the left atrial area. Significant recurrent MR was defined as grade ≥3+ at 1 year.

### Follow-Up and End Points

After surgery, patients were maintained on guideline-directed medical therapy including angiotensin-converting enzyme inhibitors or angiotensin II receptor blockers, beta-blockers, and diuretics. The primary end point was cumulative overall survival. Secondary end points were freedom from death or readmission for heart failure, and postoperative changes in MR grade, LV end-diastolic volume index (LVEDVI), and LVEF at 1 year. Heart failure readmission was defined as any hospitalization attributable to decompensated heart failure after surgery. Follow-up was complete in all 341 patients (100%) with a median duration of 4.5 years (interquartile range [IQR], 2.4-7.3 years).

### Statistical Analysis

Continuous variables were tested for normality by the Shapiro-Wilk test and expressed as mean ± SD or median (IQR) as appropriate. Normally distributed variables were compared by Student *t* test; nonnormally distributed variables by the Wilcoxon rank-sum test. Categorical variables were compared by χ^2^ or Fisher exact test. Postoperative changes in LVEDVI and LVEF were assessed with paired *t* tests. Cumulative survival and freedom from death or heart failure readmission were estimated by the Kaplan-Meier method and compared by log-rank test. Cox proportional hazard analysis was used to identify independent predictors of survival. The multivariable model was analyzed with significant predictors depicted in the univariable model. Results are expressed as hazard ratios with 95% CI. All analyses were performed with JMP version 13 (SAS Institute Inc).

## Results

### Patients

Baseline characteristics are summarized in Table 1. Patients in the CABG + MVr group had substantially higher preoperative surgical risk, as reflected by a higher median logistic European System for Cardiac Operative Risk Evaluation II (6.7% vs 2.9%; *P* < .001), greater prevalence of New York Heart Association class III or IV symptoms (54.6% vs 37.3%; *P* = .003), higher diuretic use (65.7% vs 46.8%; *P* = .001), and larger LV dimensions (median LVEDVI: 117 vs 105 mL/m^2^; *P* = .002). A history of prior percutaneous coronary intervention was more frequent in the MVr group (40.0% vs 24.9%; *P* = .006). Despite these differences, rates of complete revascularization (78.7% vs 81.5%; *P* = .54) and number of distal anastomoses did not differ significantly between groups. Off-pump CABG was performed in 35.2% of the CABG-alone group and was not used in the MVr group.

**Table 1:** Patient characteristics and surgical data

Variables	All patients		
	CABG with MV repair for moderate MR	CABG alone for mild or less MR	*P* value
	(n = 108)	(n = 233)	
Age (y)	69 (63-75)	67 (60-74)	.086
Male	82 (75.9)	208 (89.3)	.002
Logistic EuroSCORE	6.7 (4.09-11.9)	2.9 (1.67-5.15)	<.0001
NYHA functional class III or IV	59 (54.6)	87 (37.3)	.0028
Inotropic administration	10 (9.3)	10 (4.3)	.0792
Unscheduled IABP usage	6 (5.6)	17 (7.3)	.5445
Mechanical ventilation	3 (2.9)	4 (1.7)	.484
Redo surgery	3 (2.8)	7 (3.0)	.908
Hypertension	64 (59.3)	144 (61.8)	.655
Dyslipidemia	46 (42.6)	122 (52.4)	.093
Diabetes	59 (54.6)	146 (62.7)	.160
Chronic kidney disease stage 4 or 5	21 (19.4)	38 (16.3)	.480
Dialysis	11 (10.2)	16 (6.9)	.301
Atrial fibrillation	18 (16.7)	23 (9.9)	.080
Ventricular arrhythmia	4 (3.7)	12 (5.2)	.549
Cerebrovascular disease	19 (17.6)	24 (10.3)	.065
Peripheral artery disease	12 (11.1)	34 (14.6)	.347
Oral medication			
STATIN	52 (48.1)	125 (53.6)	.344
Angiotensin-converting-enzyme inhibitor or angiotensin II receptor blocker	67 (62.0)	131 (56.2)	.310
Beta-blocker	59 (54.6)	111 (47.6)	.230
Nitroglycerin	42 (38.9)	93 (39.9)	.857
Diuretics	71 (65.7)	109 (46.8)	.001
Triple-vessel disease	73 (67.6)	180 (77.3)	.061
Left main lesion	14 (13.0)	38 (16.3)	.483
Previous percutaneous coronary intervention	43 (40.0)	58 (24.9)	.006
Echocardiographic data			
Left ventricular end-diastolic dimension,	61 (56-66)	58 (54-65)	.017
Left ventricular end-diastolic volume index	117 (99-134)	105 (88-124)	.002
Left ventricular end-systolic dimension	51 (46-55)	49 (44-55)	.234
Left ventricular end-systolic volume index	79 (61-92)	71 (54-87)	.118
Left ventricular ejection fraction	32 (26-38)	33 (27-37)	.413
Left atrial dimension	43 (40-48)	40 (37-44)	<.0001
Mitral regurgitation grade			<.0001
None		45 (19.3)	
Trivial		66 (28.3)	
Mild		122 (52.4)	
Moderate	108 (100)		
Severe			
Tricuspid regurgitation grade			<.0001
None	11 (10.2)	97 (41.4)	
Trivial	42 (38.9)	111 (47.8)	
Mild	39 (36.1)	23 (9.9)	
Moderate	15 (13.9)	1 (0.4)	
Severe	1 (0.9)	1 (0.4)	
Surgery after 2010	56 (51.9)	114 (48.9)	.615

Values are presented as median (interquartile range) or n (%). *CABG*, Coronary artery bypass grafting; *MV*, mitral valve; *MR*, mitral regurgitation; *EuroSCORE*, European System for Cardiac Operative Risk Evaluation; *NYHA*, New York Heart Association; *IABP*, intra-aortic balloon pump.

### Early and Long-Term Survival

Thirty-day mortality did not differ between groups (CABG + MVr, 1.9% [n = 2] vs CABG alone, 2.1% [n = 5]; *P* = .88). During follow-up, 37 patients (34.3%) in the CABG + MVr group and 69 (29.6%) in the CABG-alone group died. Cumulative survival at 1, 5, and 10 years was 91%, 73%, and 53% in the CABG + MVr group and 92%, 77%, and 58% in the CABG-alone group, respectively (log-rank *P* = .25) (Figure 1, *A*). The rates of freedom from death or readmission for heart failure also did not differ between groups (log-rank *P* = .24) (Figure 1, *B*). The most common cause of death was cardiac-related in both groups (10.2% vs 7.3%), followed by malignancy and infectious diseases. On multivariable Cox proportional hazard analysis, advanced age, New York Heart Association class III or IV, chronic kidney disease stage 4 or 5, and peripheral artery disease were independently associated with worse survival, whereas concomitant mitral annuloplasty was not (hazard ratio, 1.27; 95% CI, 0.85-1.91; *P* = .24) (Table 2).

**Figure 1:**
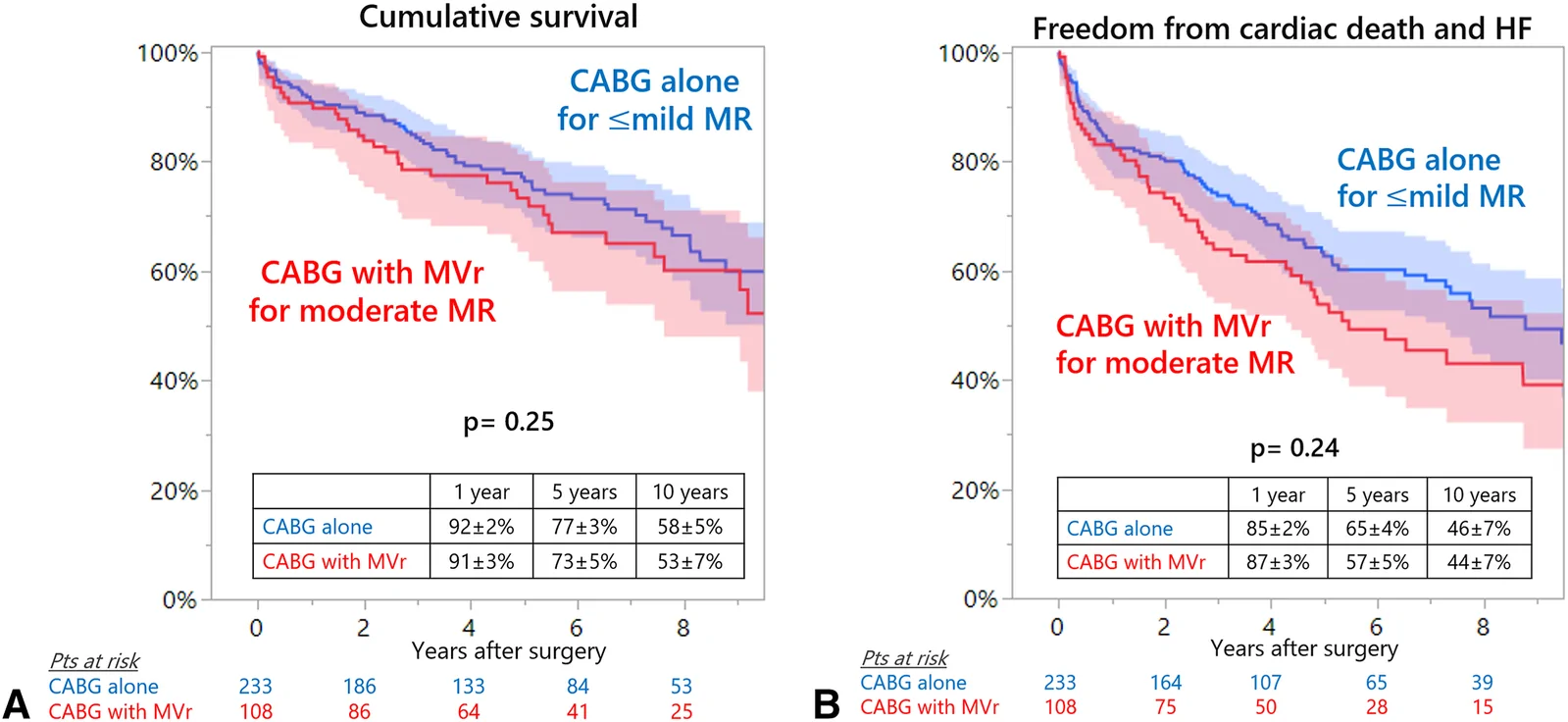
Kaplan-Meier curves for overall survival (A) and freedom from death or readmission for heart failure (B) in the entire cohort (N = 341), with 95% CI (*shaded areas*). Log-rank *P* values are shown. *CABG*, Coronary artery bypass grafting; *MVr*, mitral valve repair; *MR*, mitral regurgitation.

**Table 2:** Logistic regression analysis for cumulative survival

Variable	Univariable			Multivariable		
	Hazard ratio	95% CI	*P* value	Hazard ratio	95% CI	*P* value
Age	1.06	1.03-1.08	<.0001	1.04	1.02-1.07	.0001
Male	0.67	0.91-2.42	.1133			
NYHA functional class III or IV	1.83	1.25-2.69	.0021	1.65	1.11-2.44	.0132
Redo surgery	1.48	0.55-4.04	.4399			
Hypertension	1.44	0.95-2.19	.0889			
Diabetes	1.68	1.11-2.55	.0151	1.46	0.95-2.25	.0856
Chronic kidney disease stage 4 or 5	2.96	1.94-4.50	<.0001	2.32	1.48-3.61	.0002
Atrial fibrillation	0.93	0.49-1.72	.8123			
Cerebrovascular disease	1.56	0.85-2.88	.1543			
Peripheral artery disease	2.03	1.29-3.18	.0021	1.77	1.11-2.84	.0172
Triple-vessel disease	0.84	0.55-1.30	.4337			
Left main lesion	1.02	0.62-1.67	.9478			
Previous percutaneous coronary intervention	0.95	0.63-1.44	.8152			
Echocardiographic data						
Left ventricular end-diastolic dimension	0.98	0.95-1.01	.2112			
Left ventricular end-diastolic volume index	1.00	0.99-1.01	.9114			
Left ventricular end-systolic dimension	0.98	0.96-1.00	.1446			
Left ventricular end-diastolic volume index	0.99	0.99-1.01	.5349			
Left ventricular ejection fraction	1.00	0.98-1.03	.8053			
Left atrial dimension	0.99	0.96-1.03	.6881			
Moderate or greater mitral regurgitation	1.27	0.85-1.91	.2424			
Moderate or greater tricuspid regurgitation	0.75	0.27-2.03	.5651			
Surgery after 2010	0.73	0.46-1.11	.1329			
Off-pump CABG	1.16	0.75-1.80	.4924			
Arteries bypassed	0.92	0.77-1.10	.3460			
All arterial grafting	0.95	0.63-1.42	.7922			
Complete revascularization	0.62	0.42-0.92	.0180	0.68	0.44-1.06	.0882
Concomitant mitral annuloplasty	1.27	0.85-1.91	.2426			
Concomitant tricuspid annuloplasty	1.26	0.70-2.27	.4373			
Concomitant Af ablation	0.49	0.12-1.99	.3182			

*NYHA*, New York Heart Association; *CABG*, coronary artery bypass grafting; *Af*, atrial fibrillation.

### Postoperative MR and EF

At 1 year, the prevalence of significant MR (grade ≥3+) was lower in the CABG + MVr group than in the CABG-alone group (6% [4 out of 67] vs 13% [12 out of 94]; *P* = .032) (Figure 2, *A*). Preoperative LVEF was similar between groups (32% ± 9% vs 32% ± 7%; *P* = .41). At 1 year, LVEF was significantly higher in the CABG + MVr group (46% ± 13% vs 42% ± 12%; *P* = .044) (Figure 2, *B*). At 3 years, the prevalence of significant MR was 4% [2 out of 52] in the CABG + MVr group.

**Figure 2:**
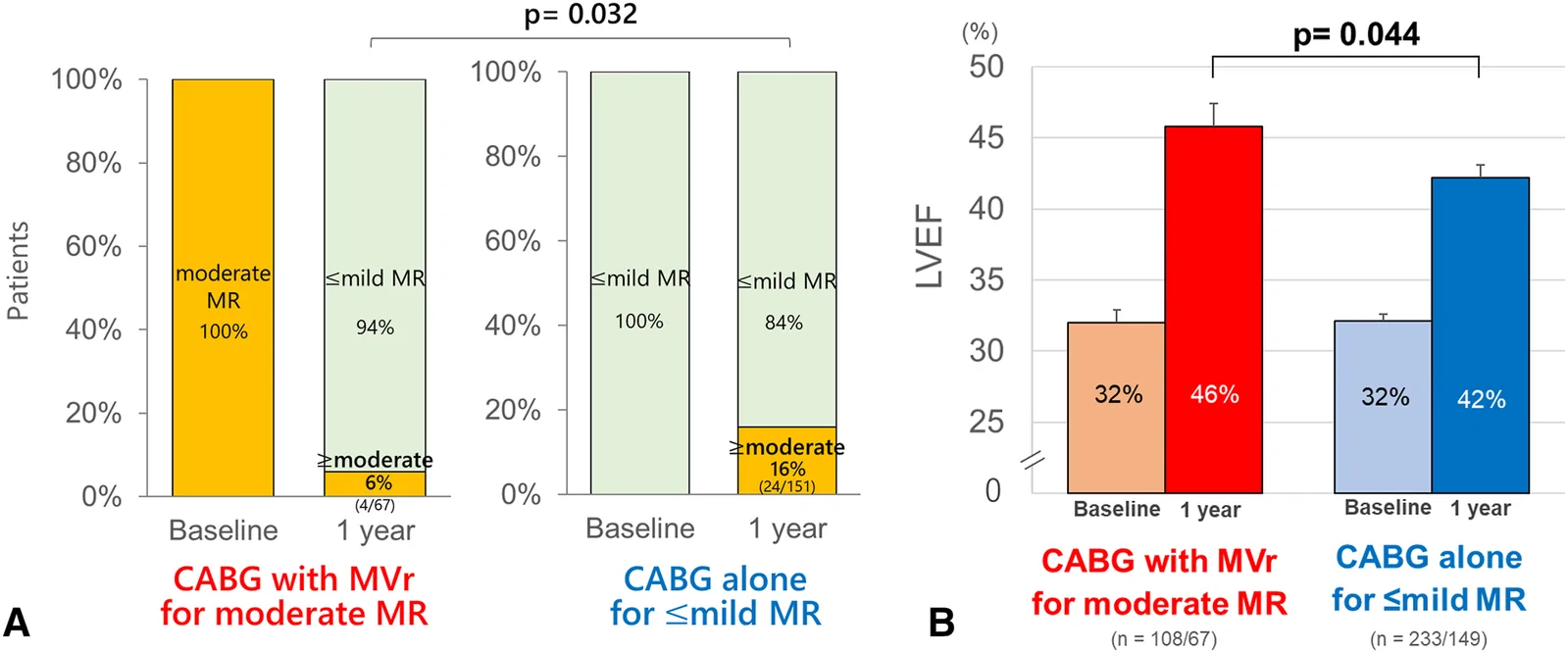
Pre- and postoperative mitral regurgitation (*MR*) grade (A) and left ventricular ejection fraction (*LVEF*) (B) in each group. Significant MR was defined as grade ≥3+. *P* value by χ^2^ test (A) and paired *t* test (B). *CABG*, Coronary artery bypass grafting; *MVr*, mitral valve repair.

### LV Reverse Remodeling

Preoperative LVEDVI was significantly larger in the CABG + MVr group (117 ± 24 vs 107 ± 27 mL/m^2^; *P* = .002) (Figure 3, *A*). At 1 year, LVEDVI was comparable between groups (85 ± 26 vs 89 ± 26 mL/m^2^; *P* = .40). The CABG + MVr group demonstrated significantly greater reduction in LVEDVI compared with the CABG-alone group, both in absolute terms (median, −30 mL/m^2^ [IQR, −47 to −15 mL/m^2^] vs −19 mL/m^2^ [IQR, −35 to −4 mL/m^2^]; *P* = .001) and as a percentage of baseline (−26% [IQR −40% to −14%] vs −19% [IQR −30% to −4%]; *P* = .001) (Figure 3, *B*).

**Figure 3:**
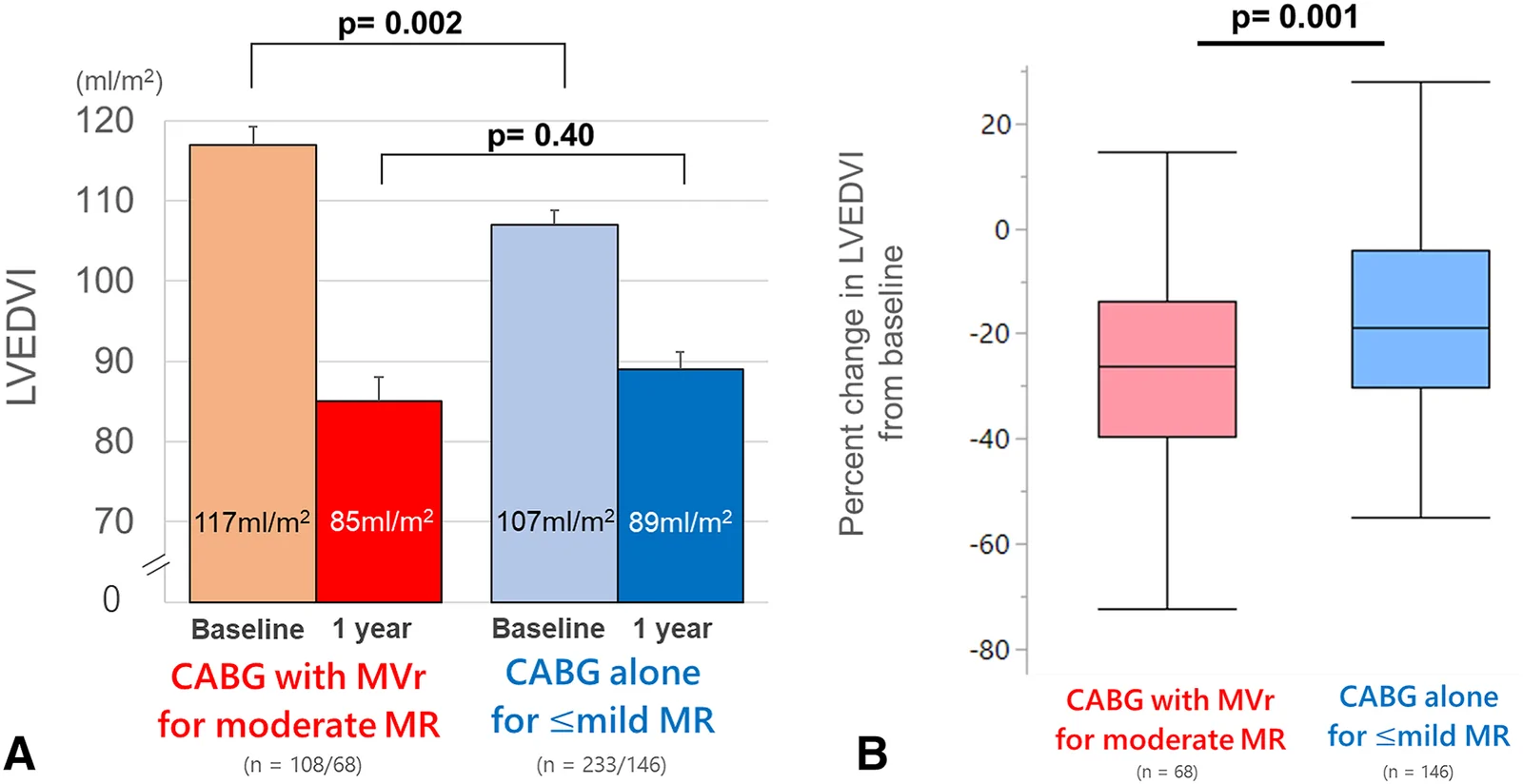
A, Pre- and postoperative left ventricular end-diastolic volume index (*LVEDVI*) in each group. B, Box plots showing percent changes in LVEDVI at 1 year after surgery in the coronary artery bypass grafting (*CABG*) plus miral valve repair (*MVr*) group versus the CABG-alone group. The box spans 25th to 75th percentiles; the *center line* denotes the median; whiskers denote 5th-95th percentiles. *P* value by Wilcoxon rank-sum test.

## Discussion

This study compared long-term outcomes of concomitant MVr for moderate ischemic MR versus CABG alone in patients with mild or absent MR, all with severely impaired LV function. Three principal findings merit emphasis. First, patients with moderate MR who underwent CABG + MVr carried substantially higher preoperative risk than those undergoing CABG alone for nonsignificant MR. Second, despite this risk differential, early and late mortality were comparable between groups. Third, the CABG + MVr group achieved significantly greater LV reverse remodeling and superior MR control at 1 year, with postoperative LVEDVI converging toward that of the CABG-alone group (Figure 4).

**Figure 4:**
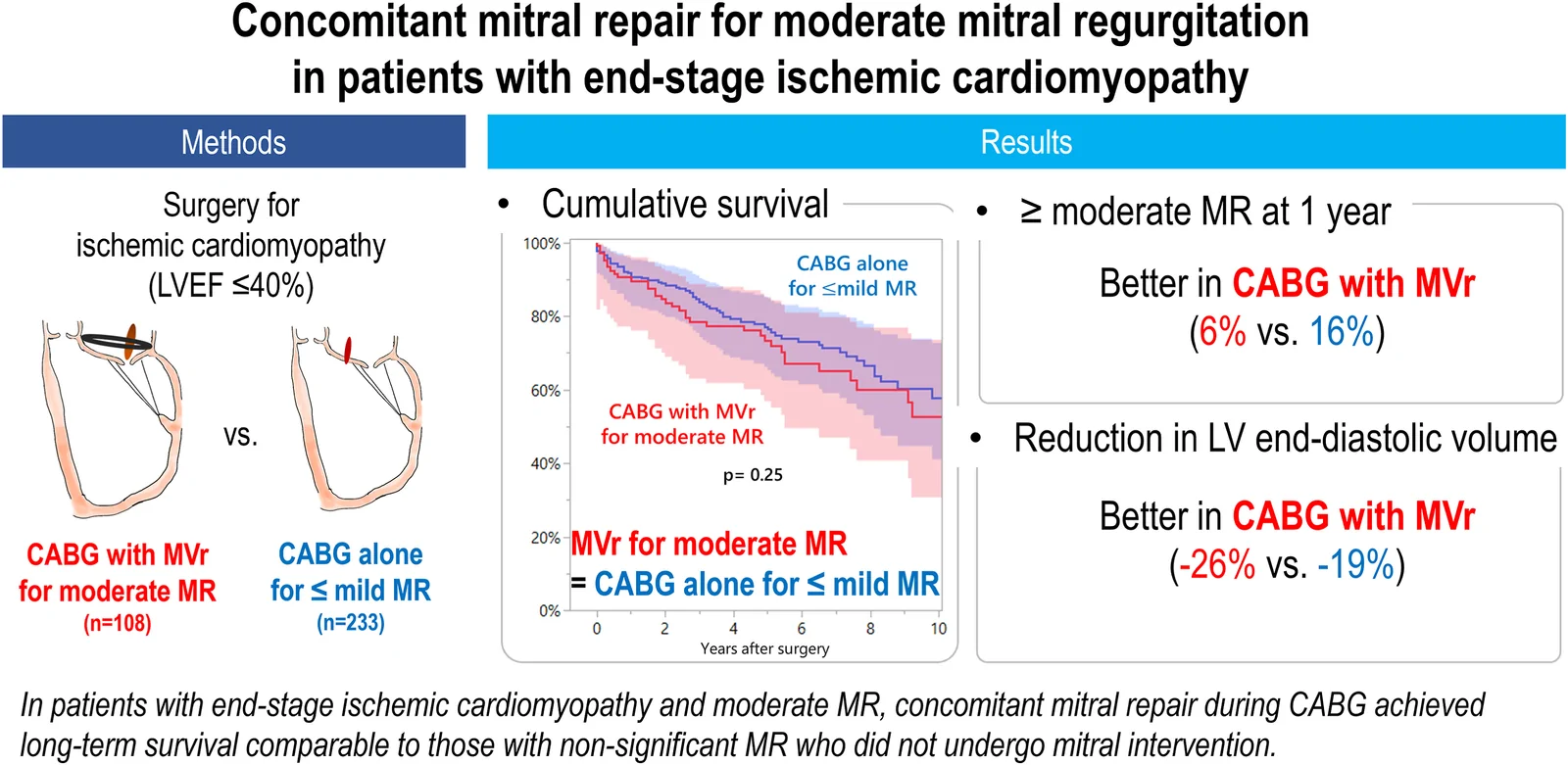
Graphical abstract depicting the study's methods, results, and clinical implications. *CABG*, Coronary artery bypass grafting; *MR*, mitral regurgitation; *MVr*, mitral valve repair; *LV*, left ventricle.

The natural history of ischemic MR is well established as unfavorable, and even mild-grade MR carries independent adverse prognostic significance in patients with reduced LV function.^5^^,^^6^ The present study demonstrates that surgical correction of moderate MR in the highest-risk population—patients with LVEF averaging 32% and markedly enlarged ventricles—can equalize long-term prognosis with that of patients whose MR did not meet criteria for intervention. Fattouch and colleagues^15^ reported 10-year results of a Cardiothoracic Surgical Trials Network randomized trial in which overall survival was equivalent between patients with moderate ischemic MR randomized to CABG + ring annuloplasty versus CABG alone. Li and colleagues^18^ similarly reported comparable survival between isolated CABG and concomitant MVr in 158 patients with moderate ischemic MR and reduced LVEF over a mean follow-up of 31 months. Compared with both prior studies, the present cohort was notably older (median age, 68 vs 60-65 years) and had more severely impaired LV function (LVEF 32% vs 37%-45%); nevertheless, MVr normalized long-term prognosis—an observation that may support a broader application of the combined approach in patients with advanced ischemic cardiomyopathy.

Coronary revascularization is a critical determinant of outcome in ischemic cardiomyopathy. Adequate revascularization of viable myocardium improves regional LV wall motion and may reduce functional MR independently.^19^ In the present study, incomplete revascularization was identified as a significant adverse prognostic factor—consistent with prior reports by Kusunose and colleagues^20^ and Cavalcante and colleagues,^21^ who similarly identified incomplete revascularization as an independent predictor of adverse outcomes in patients undergoing mitral surgery for ischemic cardiomyopathy. The comparable rates of complete revascularization between groups in the present study support the interpretation that differences in postoperative LV remodeling were predominantly driven by the addition of mitral repair rather than differences in revascularization extent.

Regarding LV reverse remodeling, our findings align with those of Chan and colleagues,^11^ who demonstrated in a randomized trial of 34 patients (LVEF, ∼40%, LV end diastolic dimension, 57 mm) that CABG + ring annuloplasty yielded greater LV dimension reduction and superior MR control compared with CABG alone at 1 year. In contrast, the Cardiothoracic Surgical Trials Network trial—enrolling patients with higher baseline LVEF and smaller ventricular volumes—did not demonstrate a significant difference in LV volume reduction between treatment arms.^14^ Li and colleagues^18^ also reported that concomitant MVr improved postoperative MR grade and showed a trend toward better LV contractility, but LV dimensions were comparable between groups. Taken together, these data suggest that patients with more severely dilated ventricles may derive greater remodeling benefit from concomitant mitral repair, likely because volume unloading has a more substantial hemodynamic influence when preload is markedly elevated.

The Cavalcante and colleagues^21^ study of 578 patients with ischemic cardiomyopathy is particularly instructive in the context of the present findings. In that cohort (LVEDVI, ∼148 mL/m^2^; LVEF, ∼28%), more severe MR, larger infarct size, and incomplete revascularization each predicted worse outcomes. Given that the present cohort had a somewhat higher LVEF (32%) and lower LVEDVI (117 mL/m^2^), infarct burden was likely less extensive, which may partly explain the favorable 5-year survival (∼77%) observed in the CABG-alone group and the comparable outcomes achieved with MVr.

### Limitations

This study has several important limitations. First, the retrospective, nonrandomized design introduces selection bias; patients assigned to MVr had more severe preoperative disease, and residual confounding cannot be excluded despite multivariable analysis. Second, the sample size limits statistical power, particularly for subgroup analyses. Third, LV volumes were assessed by 2-dimensional echocardiography, which may be less precise than cardiac magnetic resonance imaging or computed tomography. Fourth, the long study period (1995-2023) encompasses substantial changes in surgical technique, device availability, and guideline-directed medical therapy, which may introduce era-related heterogeneity. Although it is known that guideline-directed medical therapy plays an important role in postoperative cardiac function, the influence of postoperative medication could not be evaluated in this study due to the absence of such data in our database. Prospective validation in a larger, contemporaneous cohort is warranted.

## Conclusions

In patients with end-stage ischemic cardiomyopathy and moderate MR, concomitant MVr can be performed with acceptable operative mortality. After MVr, patients with moderate MR achieved long-term survival comparable to those with nonsignificant MR who did not undergo mitral intervention, along with greater LV reverse remodeling and superior MR control. These findings support consideration of concomitant mitral repair in carefully selected patients with moderate ischemic MR and advanced ventricular dysfunction.

## Conflict of Interest Statement

The authors reported no conflicts of interest.

The *Journal* policy requires editors and reviewers to disclose conflicts of interest and to decline handling or reviewing manuscripts for which they may have a conflict of interest. The editors and reviewers of this article have no conflicts of interest.

## Figures and Tables

**Figure E1:**
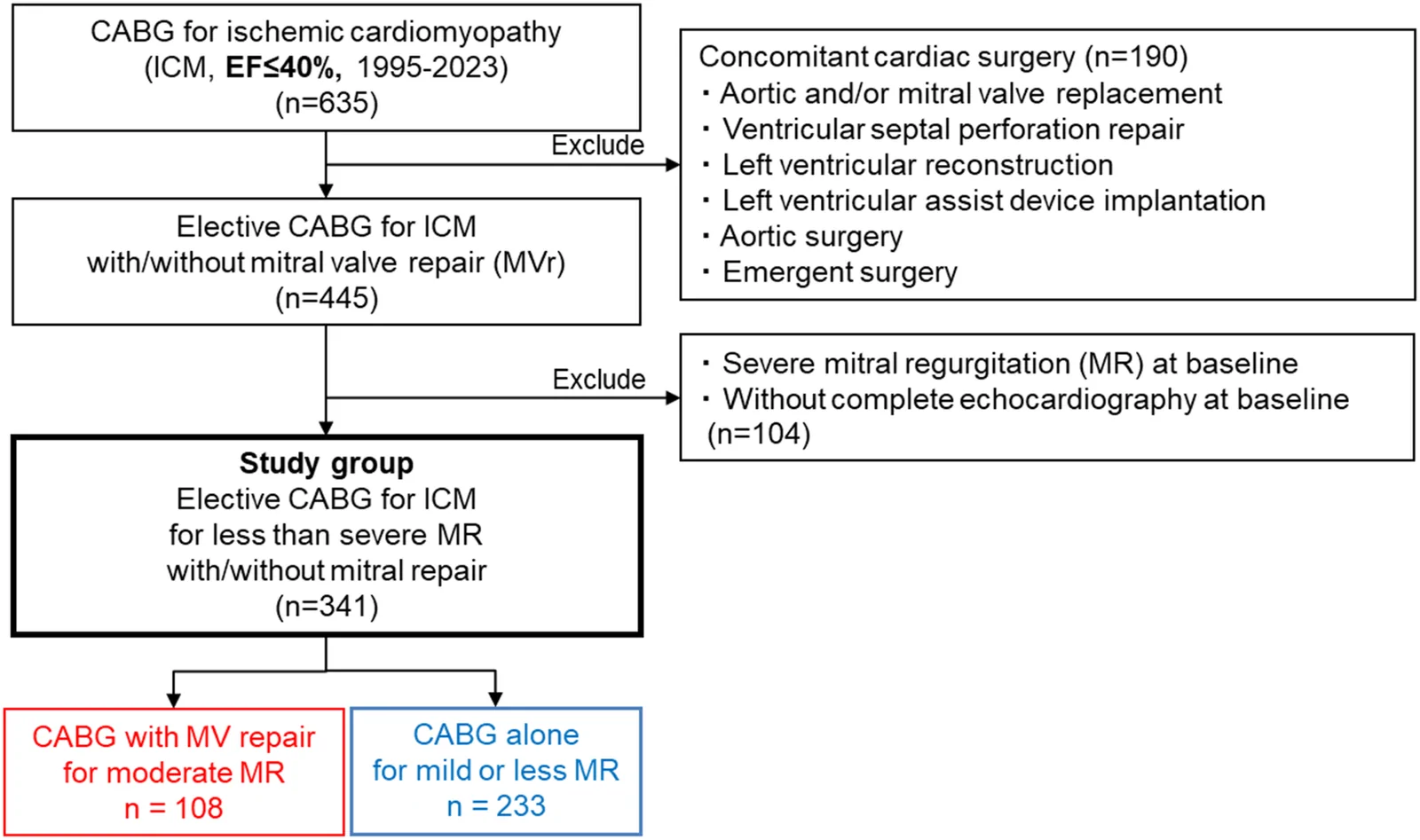
Participant flow diagram depicting patient selection. *CABG*, Coronary artery bypass grafting; *ICM*, ischemic cardiomyopathy; *EF*, ejection fraction; *MVr*, mitral valve repair; *MR*, mitral regurgitation; *MV*, mitral valve.
